# Attachment Dimensions and Infertility: Exploring Psychological Outcomes Through Systematic Review and Meta‐Analysis

**DOI:** 10.1111/jmft.70073

**Published:** 2025-09-08

**Authors:** Camilla Tacchino, Rosetta Castellano, Guyonne Rogier, Teresa Cocchiaro, Alessandro Dal Lago, Rocco Rago, Patrizia Velotti

**Affiliations:** ^1^ Department of Dynamic and Clinical Psychology, and Health Studies Sapienza University of Rome Rome Italy; ^2^ Unicamillus ‐ Saint Camillus International University of Health Sciences Rome Italy; ^3^ Department of Gender, Parenting, Child and Adolescent Medicine, Unit of Reproductive Pathophysiology and Andrology “Sandro Pertini” Hospital Rome Italy

**Keywords:** attachment, couple, distress, infertility, quality of life

## Abstract

This systematic review and meta‐analysis examines the role of romantic attachment as a protective or risk factor in how individuals cope with infertility diagnosis, treatment, and outcomes. A systematic search was conducted across six databases from January 1, 2011, to February 3, 2025. Seventeen studies met inclusion criteria, exploring associations between romantic attachment and individual psychological correlates of infertility. Seven studies examining links between attachment (anxiety and avoidance) and infertility‐related stress were included in a meta‐analysis. Findings highlight significant associations between insecure attachment and various psychological outcomes, including infertility‐related stress, general well‐being, mental health symptoms, and body image concerns. Meta‐analytic results confirmed moderate, positive associations between both attachment anxiety and avoidance and infertility‐related stress. The review underscores the influence of attachment in couple relationships on coping with infertility, emphasizing the distinct roles of attachment anxiety and avoidance. These insights offer valuable clinical implications.

**Trial registration:** PROSPERO (CRD42024523311).

## 1 Introduction

### 1.1 Psychological Impact of Infertility

Global estimates indicate that approximately 15%–20% of couples attempting to conceive experience reproductive challenges (Babul‐Hirji et al. 2021). The increasing prevalence of these difficulties has been recognized as a growing public health concern (World Health Organization 2024). A substantial body of research has investigated the psychological impact associated with various stages of this experience, from diagnosis and treatment processes, including assisted reproductive technologies (ARTs) (Vioreanu 2021), to the transition into parenthood following treatment (Hammarberg et al. 2008), and the long‐term adaptation to involuntary childlessness (Gameiro and Finnigan 2017). These studies consistently highlight profound effects on both individual well‐being and couple dynamics.

The diagnostic phase often follows prolonged and invasive medical evaluations, which can trigger emotional responses such as disbelief, denial, self‐blame, frustration, social withdrawal, feelings of injustice, and helplessness (Sezgin and Hocaoglu 2014). ART treatments involve considerable physical, emotional, financial, and temporal demands (Riccio 2017) and are regarded as among the most stressful life events, second only to the loss of a loved one or relationship breakdown (Wiweko et al. 2017). Additionally, the uncertainty of treatment outcomes exposes individuals to the persistent risk of failure (Kushnir et al. 2017), which can adversely affect self‐esteem, foster isolation, and, in some cases, lead to discontinuation of treatment (Shafaghi et al. 2024). Emotional distress is common; for instance, Yusuf (2016) reported that 29% of women experiencing infertility exhibited severe stress symptoms. Common sources of distress include relational and social concerns, encompassing difficulties in maintaining intimacy within the couple and a perceived sense of disconnection from peers who have children, as well as the centrality of parenthood within the couple's shared life goals, and the emotional burden associated with confronting the prospect of a future without the possibility of becoming parents (Newton et al. 1999).

Research has also highlighted gender differences in psychological responses. Women frequently report elevated levels of general psychopathology, including anxiety, depression, lowered self‐esteem, and heightened perceptions of social stigma, which can engender feelings of incompleteness and failure (Ying et al. 2015). Men with male‐factor infertility often experience reduced quality of life and increased infertility‐related stress, as the condition may be perceived as a threat to masculinity and virility (Biggs et al. 2024). Despite these gender‐specific manifestations, infertility is widely acknowledged as a deeply stressful event for both partners, negatively impacting individual well‐being and couple quality of life. Predictors of impaired mental health across genders include lower educational attainment and previous unsuccessful IVF attempts. Specific risk factors for women include dissatisfaction with sexual life, refusal to attend counseling, and having a partner who feels sexually pressured; for men, risk factors encompass a strong desire for parenthood, poor marital relationship quality, and longer duration of reproductive challenges (Chachamovich et al. 2010).

A meta‐analysis by Fallahzadeh et al. (2019) confirmed that women facing these challenges exhibit higher levels of depression compared with fertile counterparts. Depressive symptoms and a sense of loss often stem from fears of being unable to have genetically related children and repeated treatment failures (Swanson and Braverman 2021). Notably, depression may influence treatment decisions, with women screening positive for depressive symptoms less likely to pursue oral medications or IVF (Crawford et al. 2017). Anxiety in this context is often linked to a perceived lack of control, manifesting as intrusive thoughts, persistent worry, and anxiety‐driven behaviors, such as compulsive online information seeking (Swanson and Braverman 2021). High state anxiety correlates with elevated neuroticism and lower extroversion (Shafierizi et al. 2022). Among women undergoing ART, additional anxiety risk factors include somatic symptoms and poor sleep quality (Wang et al. 2023).

Furthermore, ART treatments may profoundly affect individuals' relationships with their bodies, particularly women, due to intense hormonal stimulation, which can disrupt their sense of femininity (Cocchiaro et al. 2020). Experiences of bodily betrayal can contribute to body image avoidance and dissatisfaction with one's physical self (Salcuni et al. 2021).

The challenges encountered by couples facing reproductive issues have been widely documented in the literature, which frames this condition as a significant psychological stressor. Its impact extends to heightened levels of perceived stress, diminished quality of life, the presence of psychopathological symptoms, and a disrupted relationship with one's body. As this experience is shared within the dyad, both partners are required to mobilize personal and relational coping resources to manage its demands (García‐Quintáns et al. 2023). In this context, the attachment bond may play a pivotal role in shaping individual adjustment.

### 1.2 The Role of Attachment

Infertility often elicits profound feelings of vulnerability, dependence, and an increased need for emotional closeness and reassurance, thereby activating the attachment behavioral system (Lowyck et al. 2009). According to attachment theory, romantic partners in adulthood typically serve as primary attachment figures, individuals to whom one turns in times of emotional distress (Shaver and Mikulincer 2009). Within this theoretical framework, the quality of romantic attachment functions as either a protective or risk factor when coping with major life stressors, such as infertility (Castellano et al. 2014). Secure attachment styles are generally associated with enhanced emotional regulation and relational resilience (Mikulincer and Shaver 2016; Simpson and Rholes 2017), whereas insecure styles—namely, anxious and avoidant—may exacerbate psychological distress and interpersonal strain during the infertility experience (Mikulincer et al. 1998).

In the present paper, consistent with the conceptualization most commonly employed in primary studies (Calvo et al. 2023; Péloquin et al. 2024; Qu et al. 2024), we focus on insecure attachment within couple relationships as comprising two key dimensions: anxiety and avoidance (Hazan and Shaver 1987). Individuals scoring high on attachment anxiety tend to be preoccupied with their relationships, seeking constant reassurance and fearing abandonment; conversely, those high in attachment avoidance typically experience discomfort with emotional closeness and strive for independence, often rejecting emotional reliance on others (Shaver and Mikulincer 2009).

Despite this well‐established theoretical framework, the specific contributions of attachment dimensions, namely, anxiety and avoidance, to psychological outcomes in the context of infertility remain inconclusive. While several studies have documented detrimental effects of both attachment anxiety and avoidance among individuals coping with reproductive challenges (Iordăchescu et al. 2022; Calvo et al. 2023), other research has reported significant associations exclusively with attachment anxiety (Skvirsky et al. 2019) or solely with avoidance (Moura‐Ramos et al. 2017). This variability highlights the need for a comprehensive synthesis of the existing literature. To date, no systematic review has been conducted to elucidate the role of attachment styles in the psychological experiences of individuals facing infertility.

### 1.3 The Current Study

Given the pivotal role of attachment in coping with infertility, the present study aims to conduct a systematic review and meta‐analysis of empirical research examining the associations between attachment styles within couple relationships and psychological outcomes related to infertility, with a particular focus on individual‐level effects. Systematic reviews represent a rigorous approach to synthesizing evidence by comprehensively identifying, evaluating, and critically summarizing available research on a specific topic according to widely accepted quality criteria. Systematic reviews follow explicit methods for literature search, study selection, and data synthesis. To meet these standards, this study adheres to the Preferred Reporting Items for Systematic Reviews and Meta‐Analyses guidelines for systematic reviews (PRISMA; Page et al. 2021), which provide a standardized framework detailing sources, processes, and criteria to systematically locate, select, and analyze primary studies. By synthesizing findings from the past 15 years, this review aims to offer a comprehensive and up‐to‐date overview of the literature on romantic attachment and psychological outcomes in the context of infertility, thereby acknowledging and reflecting the evolving nature of this study field.

## 2 Methods

**Figure 1:**
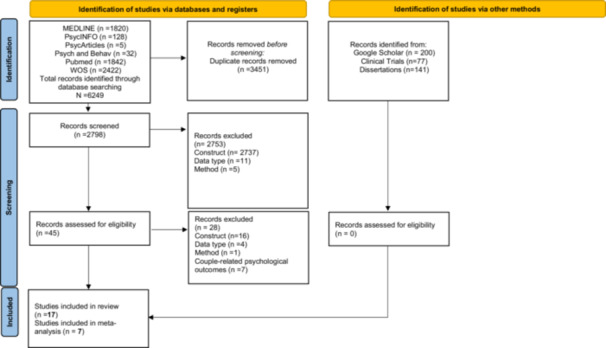
Flow diagram. [Color figure can be viewed at wileyonlinelibrary.com]

The study was performed according to the PRISMA guidelines for systematic reviews. The review was recorded on PROSPERO (No. CRD42024523311). The comprehensive process of study selection is shown in Figure 1.

### 2.1 Eligibility Criteria

The identification of eligible studies was guided by the inclusion and exclusion criteria presented in Table 1. Only studies explicitly targeting individuals or couples experiencing infertility were considered for inclusion. Eligibility was determined based on the following criteria: (1) the investigation of both romantic attachment and individual psychological outcomes in infertile participants using reliable and validated instruments; (2) the assessment of the relationship between romantic attachment and individual psychological outcomes in infertile participants and/or the comparison of psychological outcomes across infertile groups differing in attachment characteristics; (3) the nature of the contribution, specifically original empirical research; and (4) the adoption of a quantitative research design, including cross‐sectional, longitudinal, or experimental methodologies.

**Table 1:** Eligibility criteria.

	Inclusion criteria	Exclusion criteria
Construct	Measure of romantic attachment through reliable and validated measures Measure of at least one individual psychological outcome, in terms of infertility‐related stress/quality of life, general well‐being, psychopathological symptoms, relationship with one's body, in clinical participants, that is, individuals with infertility diagnosis and/or undergoing Assisted Reproductive Technology (ART) treatments, through reliable and validated measures Measure of the association between romantic attachment and psychological outcomes in clinical participants Comparison between the investigated psychological outcome levels in infertile groups with different patterns of romantic attachment	Measure of other attachment bonds (such as prenatal attachment) Measure of psychological aspects in medical conditions, which might contribute to infertility without considering the actual infertility experience (such as cancer) Measureof psychological couple‐related outcomes in infertile individuals No estimation of the association between romantic attachment and psychological outcomes and/or of differences in groups with different patterns of romantic attachment Association between romantic attachment and ART outcomes Comparison between romantic attachment styles/dimensions in groups with and without infertility problems or in different types of infertile groups
Data type	Original research	Duplicates of original research Reviews and meta‐analyses Conference proceedings
Method	Quantitative research methods Cross‐sectional Longitudinal Experimental	Qualitative research methods Free interviews Focus group Case studies Theoretical articles

### 2.2 Type of Participants

With respect to sample composition, no restrictions were applied regarding age, gender, or nationality. However, only studies involving clinical individuals undergoing an infertility‐related experience were included, based on a couple‐centered definition of infertility. Eligible participants included those who had received a personal diagnosis of infertility, whose partner had received such a diagnosis, those experiencing unexplained infertility, individuals undergoing fertility assessments after at least 1 year of unprotected intercourse without conception, or those undergoing assisted ART procedures. Importantly, studies addressing psychological aspects in medical conditions potentially associated with infertility (e.g., cancer), but not involving a currently experienced infertility condition, were excluded.

### 2.3 Type of Comparison and Outcome

Concerning the assessment of romantic attachment, all studies recurring to a recognized measurement tool were included. With regard to psychological outcomes, this review specifically focused on individual‐level outcomes in infertile individuals, even if conceptually linked to the relational dimensions of romantic attachment. Accordingly, we included only studies examining infertility‐related stress or quality of life, general well‐being, psychopathological symptoms, and body‐related experiences, while excluding those focused on couple‐level outcomes, such as relationship satisfaction or sexual functioning. The decision to exclude studies focusing exclusively on couple‐level outcomes was based on the objective of this review to specifically examine the individual psychological impact of infertility. The aim was to explore how romantic attachment might serve as a risk or protective factor for personal distress and well‐being in this context. Although the association between attachment and relational functioning is well‐documented in the literature, the considerable heterogeneity among existing studies prompted us to focus specifically on individual‐level outcomes. This approach was intended to ensure greater conceptual coherence and to offer a clearer understanding of the personal psychological implications of the infertility experience.

Studies were included in the present review if they reported associations between romantic attachment and individual psychological outcomes, or if they examined differences in psychological outcomes across groups characterized by distinct attachment patterns. For inclusion in the meta‐analysis, studies were additionally required to report effect sizes (ESs) for the association between the constructs, either as correlation coefficients or group comparisons, along with relevant descriptive statistics. If a paper measured the relevant constructs, without reporting the ES for their association, the corresponding author was reached out to obtain the missing information. Of the three contacted authors, none provided the requested additional data. These studies were therefore included in the systematic review but were not eligible for inclusion in the meta‐analysis. Specifically, the missing indices concerned the associations between attachment and anxiety symptoms, positive mental health, and positive affect, respectively. These data were requested with the aim of reaching a sufficient number of studies for conducting separate meta‐analyses on these constructs. However, due to the lack of available data, this was not possible, and the meta‐analysis was conducted solely on the relationship between attachment and infertility‐related stress.

### 2.4 Type of Studies

To be included, studies were required to adopt a quantitative methodology. To maximize the comprehensiveness of the review, no restrictions were applied with respect to research design, language, or country of origin. Additionally, publication in a peer‐reviewed journal was not a prerequisite for inclusion. Conversely, studies that did not present original empirical data, such as literature reviews, meta‐analyses, theoretical papers, or conference abstracts, were excluded.

### 2.5 Search Strategy

The following databases were consulted for a literature search of the last 15 years (from January 1, 2011, to February 3, 2025): MEDLINE, PsycINFO, PsycArticles, Psychology and Behavioral Sciences Collection, PubMed, and Web of Science. The research filter, available in Supporting Information Appendix A, was composed of two main concepts, respectively, related to romantic attachment and infertility. The research was conducted using the title/abstract field codes. Later, we examined the gray literature to detect eligible works, consulting the first 200 results from Google Scholar (Haddaway et al. 2015), systematically searching the ClinicalTrials.gov registry for ongoing or unpublished studies, and examining the EBSCO Open Dissertations database for relevant theses and dissertations on the topic.

### 2.6 Study Selection

The study selection process was independently conducted by two authors. The initial database search retrieved 6249 records. After removing 3451 duplicates, 2798 studies were screened based on title and abstract. At this stage, studies were excluded for the following reasons: lack of alignment with the constructs of interest (*n* = 2737), nonoriginal data types (*n* = 11), or methodological reasons (*n* = 5). This initial screening resulted in 45 full‐text articles being assessed for eligibility. Of these, 28 were excluded due to construct‐related mismatch (*n* = 16), inappropriate data type (*n* = 4), methodological issues (*n* = 1), or focus on couple‐related rather than individual psychological outcomes (*n* = 7). A parallel search of gray literature sources, including Google Scholar, ClinicalTrials.gov, and EBSCO Open Dissertations, yielded 418 additional records, none of which met the inclusion criteria after screening. Ultimately, 17 studies were included in the systematic review, with 7 eligible for the meta‐analysis. This selection process is illustrated in the PRISMA flow diagram (Figure 1).

### 2.7 Data Extraction Process

A standardized protocol was employed to extract and code data across the following categories: (1) study information (authors, year, publication status, and country), (2) sample characteristics (sample size, gender, age, and composition), (3) methodological features (research design, targeted constructs, measurement instruments, and quality assessment), and (4) main findings (including relevant statistical indices). Studies were then categorized into four thematic strands: (1) investigations of the relationship between attachment and infertility‐related stress or quality of life, (2) analyses of the association between attachment and general well‐being, (3) research on the link between attachment and psychopathological symptoms, and (4) studies examining the connection between attachment and body‐related experiences. Extracted data are presented in detail in Supporting Information Appendix B.

The meta‐analysis was conducted exclusively on studies examining the association between romantic attachment, distinguishing attachment anxiety and avoidance, and infertility‐related stress, as assessed by the Fertility Problem Inventory (FPI; Newton et al. 1999). Meta‐analytic procedures followed the recommendation that a minimum of five independent studies investigating the same constructs is required to ensure meaningful synthesis (Myung 2023). Subsequently, moderator analyses were performed to evaluate potential influences on the relationship between attachment dimensions and infertility‐related stress. Specifically, moderators included gender (female vs. male), mean sample age, country of study conduction (Italy vs. other), and study quality. Moderation analyses were limited to these variables due to sufficient variability and lack of excessive homogeneity within the dataset.

### 2.8 Assessment of Methodological Quality

**Figure 2:**
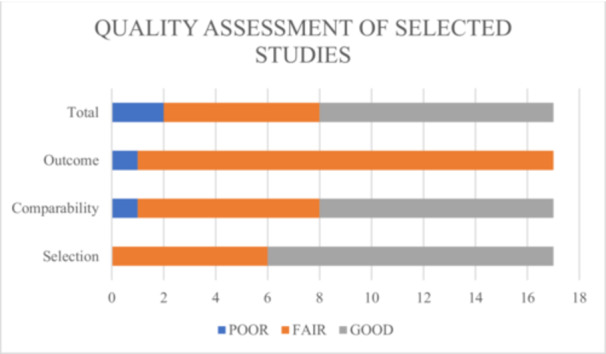
Qualitative assessment of selected studies. [Color figure can be viewed at wileyonlinelibrary.com]

The quality of studies was evaluated through an adapted version of the Newcastle–Ottawa Scale (Wells et al. 2000; Modesti et al. 2016), tailored for cross‐sectional works. Two authors independently assessed the quality of the studies. Any discrepancies were resolved through discussion and, when necessary, consultation with a third reviewer (see Figure 2).

### 2.9 Statistical Analyses

Two separate meta‐analyses were conducted to examine (a) the relationship between attachment anxiety and infertility‐related stress, and (b) the relationship between attachment avoidance and infertility‐related stress. The Comprehensive Meta‐Analysis software (CMA, version 3.7) was used to calculate the combined ES from the individual ESs reported in each study. Specifically, Pearson's *r* correlation coefficients served as the ES measure to assess the associations between attachment dimensions (anxiety and avoidance) and overall infertility‐related stress, as measured by the FPI. The exception was the study by Moura‐Ramos et al. (2017), which focused solely on the need for parenthood subscale of the FPI. No transformations of statistical metrics were necessary, as all included primary studies directly reported correlation coefficients (*r*).

### 2.10 Calculation of ESs

The ESs were calculated using a random effects model (Rosenthal 1995), which is considered more suitable when substantial variability among the ESs is present (Cooper et al. 2019). The magnitude of the overall effect and its 95% confidence interval (CI) were determined following the procedure outlined by Borenstein et al. (2021). Heterogeneity was assessed employing the *Q* statistic (Cochran 1954) and the *I* ^2^ percentage (Higgins 2003), which estimate the variance associated with the effects. Additionally, both categorical (gender and country) and continuous (age and quality assessment) moderators were investigated through a meta‐regression approach.

### 2.11 Publication Bias

Duval and Tweedie's (2000) trim‐and‐fill method was employed to calculate publication bias. The trim‐and‐fill approach by Duval and Tweedie (2000) was used to assess publication bias for each subset of the meta‐analysis, providing an estimate of the number of unpublished studies due to publication bias, as well as estimated ESs assuming no publication bias.

## 3 Results

### 3.1 Results of the Systematic Review

In total, 17 studies published over the past 15 years examining romantic attachment and individual psychological outcomes in the context of infertility were included in this review (see Supporting Information Appendix B). Of these, six studies were conducted in Italy (35%), two in Ireland (12%), two in Israel (12%), and the remaining contributions originated from Greece, Portugal, Turkey, Romania, Canada, China, and Pakistan. With the exception of the longitudinal study by McLaughlin and Cassidy (2019), all works adopted a cross‐sectional design and involved clinical samples, yielding a total of 4563 participants.

Most studies (59%) exclusively recruited infertile women, while the remaining (41%) involved heterosexual couples. Participants were predominantly recruited from fertility treatment centers. However, Iordăchescu et al. (2022) and Calvo et al. (2023) recruited participants, women with either a female‐factor diagnosis or experiencing any form of infertility, via social media platforms. Among the studies recruiting from ART centers, most specified the inclusion criterion as attending a fertility clinic due to infertility (Moura‐Ramos et al. 2017; Shlomo et al. 2019; Skvirsky et al. 2019; Kalci et al. 2020; Péloquin et al. 2024), while others required that participants had initiated fertility procedures (Donarelli et al. 2016; Molgora et al. 2020), or were actively undergoing ART treatments at the time of recruitment (Cassidy 2016; Theodoridou et al. 2016; Renzi et al. 2020). Some studies adopted more specific inclusion criteria, such as having initiated their first ART procedure (Donarelli et al. 2012; Salcuni et al. 2021), undergoing ART treatment for more than 2 years (Mobeen and Dawood 2023), or receiving artificial insemination with donor sperm (Qu et al. 2024). The longitudinal study by McLaughlin and Cassidy (2019) collected data at two time points: during an IVF cycle and again 1 year later.

The included studies were organized into four thematic strands, with some contributions addressing multiple psychological domains and thus being included in more than one category. Specifically, 11 studies examined the relationship between romantic attachment and infertility‐related stress or quality of life, four explored links between attachment and general well‐being, seven focused on associations between attachment and psychopathological symptoms, and two investigated the connection between attachment and the relationship with one's body.

Meta‐analyses were performed only when a sufficient number of studies (minimum of five) were available—specifically, those assessing the association between attachment anxiety/avoidance and infertility‐related stress as measured by the FPI. The FPI conceptualizes infertility stress across five dimensions: social concerns (e.g., sensitivity to comments and social isolation), sexual concerns (e.g., reduced enjoyment and scheduled intercourse), relationship concerns (e.g., communication difficulties and fears about relationship stability), need for parenthood (e.g., parenthood as a central life goal), and rejection of a childfree lifestyle (e.g., negative attitudes toward living without children).

The subsequent sections present first the qualitative synthesis of the included studies, followed by the results of the meta‐analyses.

### 3.2 Attachment and Infertility‐Related Stress/Quality of Life

Eleven studies analyzed the associations between romantic attachment and aspects of distress or quality of life, assessed with tools evaluating these constructs with specific reference to the infertility condition. More specifically, attachment was primarily evaluated using the Experiences in Close Relationships Scale (ECR; Brennan et al. 1998), which defines attachment in terms of the two dimensions of anxiety about abandonment and avoidance of closeness, except for the study by Moura‐Ramos et al. (2017), which employed the Adult Attachment Scale‐Revised (AAS‐R; Collins 1996), similarly providing scores for attachment anxiety and avoidance. Regarding the outcomes included in this strand, they comprised infertility stress, as operationalized by the FPI, covering dimensions such as social, relational, and sexual concerns, need for parenthood, and rejection of a childfree lifestyle, which was the focus of the meta‐analysis. Additionally, this strand included the ScreenIVF dimensions (Verhaak et al. 2010), which assess feelings of helplessness, lack of acceptance, and lack of perceived social support in relation to infertility, the Difficulties Experienced Scale (Benyamini et al. 2005) evaluating distress in various areas among infertile women, and infertility‐related quality of life, measured by the Fertility Quality of Life (FertiQoL) (Boivin et al. 2011), which includes dimensions such as emotional impact (sadness, resentment, and grief), mind–body impact (e.g., fatigue or pain), relational impact (e.g., sexuality, communication, and commitment), social impact (expectations and support), and treatment effects (environment and tolerability).

Findings showed that attachment anxiety was positively associated with feelings of helplessness, lack of acceptance, and lack of perceived social support in both women and men (Molgora et al. 2020). Similarly, Theodoridou et al. (2016), in a sample of infertile women, found significant negative correlations between attachment anxiety and the emotional, relational, social, and global domains of the FertiQoL. Renzi et al. (2020) also reported that attachment anxiety was negatively associated with the total FertiQoL score and all its subscales, except for the relational domain. Attachment avoidance, in contrast, was linked only to the lack of perceived social support in men (Molgora et al. 2020), and showed a significant negative correlation with the relational subscale of the FertiQoL (Theodoridou et al. 2016). Higher levels of difficulties experienced by women in relation to infertility were associated with both anxious and avoidant attachment styles, compared with secure patterns (Iordăchescu et al. 2022). In Péloquin et al. (2024), path analyses revealed that attachment anxiety in women and attachment avoidance in men were directly associated with lower quality of life, whereas the associations involving attachment anxiety in men and attachment avoidance in women were mediated by their use of nonadaptive or adaptive emotion‐focused coping strategies, respectively.

### 3.3 Attachment and General Well‐Being

Five studies explored the link between romantic attachment and general well‐being. Attachment was measured in two contributions (Cassidy 2016; McLaughlin and Cassidy 2019) with the single‐item measure (Brennan and Shaver 1995), with the ECR‐short form in two other studies (Shlomo et al. 2019; Skvirsky et al. 2019), and with the AAS‐R in the remaining one (Moura‐Ramos et al. 2017). Well‐being was conceived in terms of positive and negative mental health (General Health Questionnaire‐12; Goldberg 1978), psychosocial well‐being (World Health Organization Quality of Life; WHOQoL‐bref; WHOQoL Group 1995), life satisfaction (Satisfaction with Life Scale; Diener et al. 1985), positive and negative affect (Watson et al. 1988), and lack of general stress (Perceived Stress Scale; Cohen et al. 1983).

Results showed that only attachment anxiety predicted lower life satisfaction (Shlomo et al. 2019) and higher perceived stress (Skvirsky et al. 2019), while the association between attachment avoidance and perceived stress was mediated by self‐disclosure to the mother (Skvirsky et al. 2019). Both anxious and avoidant attachment styles were associated with negative mental health outcomes, whereas secure attachment was linked to positive mental health (Cassidy 2016), and was found to be predictive of positive mental health even 1 year after the initial contact during IVF cycles (McLaughlin and Cassidy 2019). Although attachment orientations did not significantly predict positive affect, both attachment anxiety and avoidance were positively associated with negative affect (Shlomo et al. 2019). In the only study in this strand that included both men and women, both attachment dimensions negatively predicted psychosocial well‐being in both samples (Moura‐Ramos et al. 2017).

### 3.4 Attachment and Psychopathological Symptoms

Seven contributions focused on the connection between romantic attachment and psychopathological symptomatology. Attachment was once again assessed with ECR, with the exception of Mobeen and Dawood (2023), who employed the Attachment Style Questionnaire (Feeney et al. 1994), comprising the five factors of trust, discomfort with intimacy, relationships as secondary, need for approval, and relationship preoccupation. The psychopathological symptoms examined included: anxiety, measured with the State‐Trait Anxiety Inventory (Spielberger et al. 1983), the Brief Symptom Inventory (BSI; Derogatis 1993), and ScreenIVF; depression, assessed with BSI, ScreenIVF, Symptom Check List‐90 (SCL‐90; Derogatis 1983), somatization (BSI), and the Global Severity Index (GSI) of the Symptom Check List‐90‐revised.

Depressive symptoms were associated with attachment anxiety in both men and women in the research by Molgora et al. (2020), as well as in Mobeen and Dawood's work (2023), where anxious attachment also significantly predicted depression among the recruited women. Attachment anxiety was significantly associated with global psychopathological severity in both men and women in the study by Salcuni et al. (2021). Depressive symptoms were related to attachment avoidance only in men in Molgora et al. (2020), whereas Kalci et al. (2020) found positive correlations with both attachment anxiety and avoidance among infertile women. Moreover, attachment avoidance was positively correlated with somatization symptoms in women (Kalci et al. 2020) and with global psychopathological severity only in men (Salcuni et al. 2021). Significant positive associations also emerged between attachment insecurities (anxiety and avoidance) and state anxiety in women (Kalci et al. 2020; Iordăchescu et al. 2022), as well as in both men and women (Donarelli et al. 2012, 2016; Molgora et al. 2020). In the latter study, multiple linear regression revealed that, only among men, both attachment anxiety and avoidance significantly predicted higher levels of anxiety symptoms.

### 3.5 Attachment and the Relationship With One's Body

Two studies explored the association between romantic attachment, measured using the ECR‐Revised scale, and body image in individuals facing infertility. More specifically, the evaluated outcomes were body image avoidance, assessed with the Body Image Avoidance Questionnaire (Rosen et al. 1991), and positive body image, evaluated with the Body Appreciation Scale‐2 (Tylka and Wood‐Barcalow 2015).

Results showed a significant positive association between attachment anxiety and body image avoidance (Salcuni et al. 2021), as well as a significant predictive role of attachment anxiety on reduced positive body image in women (Calvo et al. 2023). Regarding attachment avoidance, it was associated with body image avoidance in men (Salcuni et al. 2021) and with reduced positive body image in women, through the mediating role of infertility‐related stress (Calvo et al. 2023).

### 3.6 Meta‐Analysis Results

To explore the relationship between romantic attachment and infertility‐related stress, as operationalized by the FPI, we conducted two separate meta‐analyses focusing, respectively, on attachment anxiety and attachment avoidance, as measured by the ECR and AAS. Each meta‐analysis included seven studies and 11 ES contributions, for a total of 2240 participants. The detailed results are presented in Table 2.

**Table 2:** Overall results for the associations between attachment dimensions and infertility‐stress.

	*k*	*n*	ES	95% CI	*z*	*p*
Attachment anxiety and infertility‐stress	7	2.240	0.43**	[0.36; 0.50]	10.67	< 0.001
Attachment avoidance and infertility‐stress	7	2.240	0.41**	[0.34; 0.47]	10.96	< 0.001

** Statistically significant values; *k* = number of studies; *n* = number of participants; ES = effect size (Pearson correlation coefficient); CI = 95% confidence interval; *z* = *z* value; *p* = *p* value.

### 3.7 Anxiety and Infertility‐Stress

**Figure 3:**
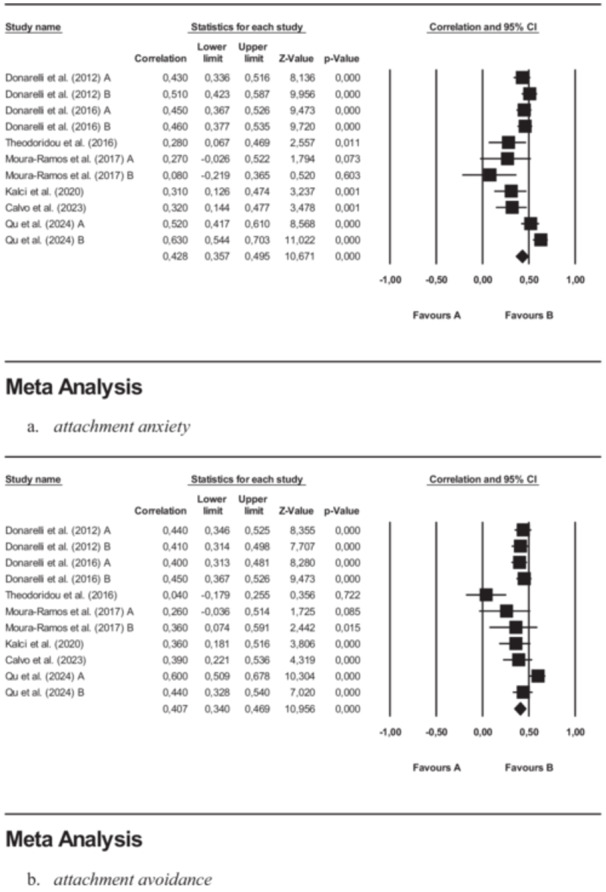
Forest plots of correlation analysis between romantic attachment and infertility‐stress: (a) attachment anxiety and (b) attachment avoidance. CI, confidence interval.

**Figure 4:**
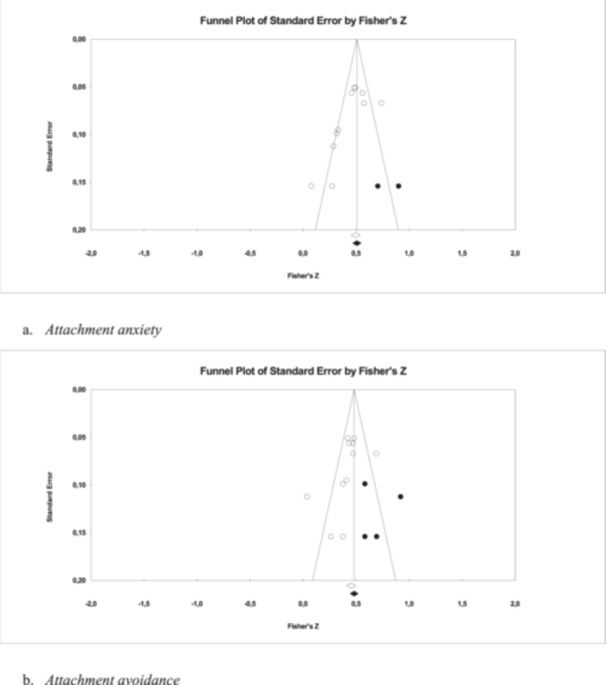
Funnel plots of publication bias analysis about infertility‐stress: (a) attachment anxiety and (b) attachment avoidance.

A significant positive mean ES of moderate magnitude (*r* 0.43, *p* < 0.001) emerged in the results regarding the relationship between attachment anxiety and infertility‐stress. Analyses showed that two studies should be added to the right size of the funnel plot and Egger's test (Egger et al. 1997) was consistently significant (CI −6.45; −0.21), confirming the presence of publication bias. This finding was adjusted using Duvall and Tweedie's trim‐and‐fill method, so that the resulting ES value was 0.45, reporting significant results (90% CI 0.38; 0.52). More information on analyses is available in forest and funnel plots in Figures 3a and 4a. These studies presented considerable heterogeneity (*Q* 35.41; *I* ^2^ = 71.76%; *p* < 0.001); therefore, we investigated the moderating role of potential covariates, the details of which are available in Table 3. Moderation analyses revealed that none of the tested moderators made significant contributions to the association between attachment anxiety and infertility‐related distress. For further details on moderation effects, see Table 3.

**Table 3:** Moderators of the relationships between romantic attachment dimensions (anxiety and avoidance) and infertility‐stress.

Moderator	Anxiety and infertility‐stress				Avoidance and infertility‐stress			
	*k*	*n*	ES	CI	*k*	*N*	ES	CI
Gender	11	2.240	0.11	[−0.05; 0.28]	11	2.240	0.04	[−0.13; 0.21]
Age	11	2.240	−0.02	[−0.05; 0.02]	11	2.240	0.03	[−0.06; 0.01]
Quality assessment	11	2.240	0.02	[−0.07; 0.11]	11	2.240	**0.09** **	[0.01; 0.16]
Country	11	2.240	−0.04	[−0.22; 0.14]	11	2.240	−0.04	[−0.20; 0.12]

** Statistically significant values. *k* = number of studies; *n* = number of participants; ES = effect size; CI = 95% confidence interval.

### 3.8 Avoidance and Infertility‐Stress

Results showed a significant positive mean ES of moderate entity (*r* 0.41, *p* < 0.001) for the association between attachment avoidance and infertility‐stress. Analyses revealed the presence of publication bias: four studies should be added on the right size of the funnel plot and Egger's test was significant (CI −5.50; 1.00). This result was modified using the trim‐and‐fill technique by Duvall and Tweedie, yielding an ES of 0.45, with significant findings (90% CI 0.38; 0.51). More details on analyses are available in forest and funnel plots in Figures 3b and 4b. These studies demonstrated significant heterogeneity (*Q* 29.71; *I* ^2^ = 66.34%; *p* < 0.001); therefore, we explored the moderating influence of potential covariates, with further details provided in Table 3. Moderation analyses highlighted a moderating effect of quality assessment. More specifically, we found that the ES tended to increase as the study quality score improved (coefficient 0.09, 95% CI 0.01; 0.16).

## 4 Discussion

Building upon the understanding that reproductive challenges constitute a significant source of stress for individuals experiencing infertility, and that such stress typically activates the attachment system (Velotti et al. 2011; Simpson and Rholes 2017), this systematic review examines studies from the past 15 years that have explored the relationship between romantic attachment and individual psychological outcomes in the context of infertility. The current review primarily includes studies conducted in European countries, which may limit the generalizability of the findings across different cultural and healthcare contexts. This geographical concentration suggests a potential sampling bias and highlights the need for future research to explore the association between romantic attachment and psychological outcomes related to infertility in more diverse populations worldwide. Notably, a substantial proportion of the studies were conducted in Italy. This may be attributed to the strong cultural emphasis traditionally placed on family within Italian society, coupled with the country's persistently low birth rates. These sociocultural and demographic factors likely contribute to increased academic and clinical interest in infertility and the use of ART.

The identified studies employed various constructs and tools and included samples with diverse gender compositions and recruitment timelines. It is important to note the prevalence of infertile women in the samples of primary studies, reflecting the broader trend in the literature where infertility research predominantly focuses on women. However, a portion of the studies recruited couples, thus including men as well. These were heterosexual couples, given the focus of the current review on infertility as clinically defined, and not on assisted reproduction related to other factors, such as the biological or physical limitations to conception encountered by LGBTQ+ populations. Therefore, the generalizability of these findings to men and other ART populations, including LGBTQ+ individuals, is limited.

This systematic review allowed the identification of four key strands examining the association between romantic attachment and psychological variables in the context of infertility, which were categorized as infertility‐related stress/quality of life, general well‐being, psychopathological symptoms, and relationship with one's body. Consistent with the results section, the discussion first addresses the findings from the systematic review, followed by those from the meta‐analysis, which specifically investigated the relationships between attachment anxiety and infertility‐related stress, and attachment avoidance and infertility‐related stress.

A primary focus of the research has been on the links between attachment dimensions and factors related to distress or quality of life in infertility, measured with context‐specific instruments. Attachment anxiety has been associated with feelings of helplessness, lack of acceptance of the infertility condition, and perceived low social support related to the infertility experience (Molgora et al. 2020), as well as with a reduced quality of life (Theodoridou et al. 2016; Renzi et al. 2020). Experiencing infertility, with its powerful connotation of defectiveness, can exacerbate negative self‐perceptions, leading to feelings of inadequacy and incompleteness (Sezgin and Hocaoglu 2014). In the context of high levels of attachment anxiety, the reported impact of infertility on one's quality of life may be linked to a tendency to magnify negative emotions and overemphasize the threatening aspects of a situation (Mikulincer and Shaver 2016).

On the other hand, attachment avoidance was associated with lower perceived social support while experiencing infertility in men (Molgora et al. 2020), and with reduced quality of life in women, particularly concerning the individually perceived impact of infertility on the couple relationship (Theodoridou et al. 2016). These findings can be interpreted in light of the typical tendencies of avoidantly attached individuals to distrust others and defensively suppress negative emotions and thoughts (Simpson and Rholes 2017). Such tendencies may hinder their ability to seek or accept adequate emotional support during ART treatments. Notably, the study by Péloquin et al. (2024) found that anxious attachment was directly associated with lower scores on the FertiQoL scale in women, although this relationship was mediated by the use of maladaptive coping strategies in men. Conversely, avoidant attachment was directly linked to lower quality of life in men, whereas in women, the association was mediated by their coping style. These findings offer valuable insight into the differential role of coping strategies adopted in response to infertility, depending on attachment characteristics and gender, with direct implications for perceived quality of life in this condition.

Another group of studies explored the association between attachment and general well‐being. The data highlighted that both anxious and avoidant attachment styles are linked to negative mental health outcomes (Cassidy 2016), greater negative affect (Shlomo et al. 2019), and lower psychosocial well‐being (Moura‐Ramos et al. 2017). Attachment anxiety, in particular, has been associated with reduced life satisfaction in women undergoing fertility treatment (Shlomo et al. 2019). More broadly, the literature has identified attachment insecurity as a significant factor contributing to diminished mental health (Zhang et al. 2022). Individuals high in attachment anxiety tend to adopt hyperactivating strategies, as persistent efforts to seek reassurance and closeness, which, although aimed at fostering connection, can paradoxically intensify emotional activation and lead to counterproductive effects (Hudson and Fraley 2018). At the same time, avoidant attachment has been associated with lower quality of life in clinical populations (Moura‐Ramos et al. 2017). Under prolonged and intense stress, the deactivating strategies typically used by avoidantly attached individuals may lose effectiveness, resulting in increased distress (Karveli et al. 2023). Furthermore, differentiated patterns emerged between the two dimensions of attachment insecurity in the study by Skvirsky et al. (2019): attachment anxiety directly predicted higher levels of general distress, whereas attachment avoidance was indirectly associated with distress through reduced self‐disclosure to the mother. Women with high levels of avoidant attachment reported less willingness to share personal emotions, which in turn is a known risk factor for increased vulnerability to stress (Mikulincer and Shaver 2016).

A set of studies focused on the association between attachment dimensions and psychopathological symptoms. The relationship between both attachment anxiety and avoidance and anxious symptomatology was widely documented (Donarelli et al. 2012, 2016; Molgora et al. 2020; Kalci et al. 2020; Iordăchescu et al. 2022). Regarding depressive symptoms, some differentiated patterns emerged: attachment anxiety was consistently associated with depressive symptoms across studies (Kalci et al. 2020; Molgora et al. 2020; Mobeen and Dawood 2023), whereas attachment avoidance was significantly associated with depressive symptoms in the female sample recruited by Kalci et al. (2020), and only in men in Molgora et al.'s study (2020). This finding aligns with the hypothesis that avoidant attachment may manifest differently across genders, with women potentially being less prone to restricting emotional expression and therefore less vulnerable to depressive outcomes (Zhang et al. 2022). Furthermore, attachment anxiety was associated with greater global psychopathological severity in both genders (Salcuni et al. 2021), while attachment avoidance was linked to somatization symptoms in women (Kalci et al. 2020) and to overall psychopathological severity in men (Salcuni et al. 2021). Prior research on adult attachment in close relationships has demonstrated that the dysfunctional relational thoughts, emotions, and behaviors typical of insecurely attached individuals are associated with greater vulnerability to psychopathological outcomes (Simpson and Rholes 2017). Individuals with high attachment anxiety typically exhibit an intense need for emotional closeness, coupled with persistent concerns about rejection or abandonment (Collins and Read 1990), thereby exacerbating individual psychological discomfort (Theodoridou et al. 2016; Molgora et al. 2020). In this regard, existing literature has highlighted how cognitive components such as perceived lack of social support and difficulty in accepting infertility are associated with the emergence of psychopathological symptoms in both women and men facing infertility (Patel et al. 2018).

A final line of research has examined the relationship between attachment dimensions and body image among individuals facing infertility. Findings indicated a significant association between attachment anxiety and both body image avoidance (Salcuni et al. 2021) and reduced positive body image in women (Calvo et al. 2023). These results align with previous studies showing that attachment anxiety is a strong predictor of negative body experiences, dissatisfaction, and low body appreciation in the general population (van den Brink et al. 2016). With regard to attachment avoidance, it was associated with body image avoidance in men (Salcuni et al. 2021)—a finding consistent with the use of emotional detachment strategies—and with reduced positive body image in women, through the mediating role of infertility‐related stress (Calvo et al. 2023). Taken together, these studies highlight the relevance of attachment orientations in understanding body image concerns and feelings of betrayal by one's own body among individuals dealing with infertility.

Regarding the meta‐analysis results, which focused on the relationship between romantic attachment and infertility‐related stress as conceptualized through the FPI, a significant positive association was found between attachment anxiety and infertility‐related stress, with a moderate ES. After adjusting for publication bias, the ES remained significant, thus corroborating findings from primary studies. These results highlight that individuals with high levels of attachment anxiety are particularly vulnerable to the psychological stress associated with infertility. Such individuals often exhibit heightened concerns about their partner's availability and support, which can intensify the perception of infertility as a threatening and uncontrollable life event. This dynamic may exacerbate existing insecurities, leading to increased emotional distress during the infertility experience (Brandão et al. 2020). Regarding attachment avoidance, the meta‐analysis revealed a significant positive ES of moderate magnitude, which slightly increased after adjustment using the trim‐and‐fill method. Moderation analyses further indicated that the strength of this association was greater in studies with higher methodological quality. These findings align with broader literature suggesting that individuals high in avoidance, characterized by discomfort with closeness and reluctance to depend on others, are less likely to express personal concerns (Collins 1996). When confronted with infertility, such individuals may adopt deactivating coping strategies that inhibit the use of attachment‐based mechanisms for distress regulation, as distressing thoughts and emotions remain unshared and unprocessed externally (Mikulincer and Shaver 2016).

### 4.1 Limitations and Future Directions

Although our study was conducted systematically and thoroughly, it is not without limitations. One primary limitation lies in the assessment of various individual psychological correlates using a wide range of measurement tools, which complicates the integration and comparison of results across different domains. Future research could help address this issue by employing more standardized measures, thereby facilitating meta‐analyses focused on specific dimensions as additional studies emerge. Moreover, the exclusion of studies focusing exclusively on couple‐level outcomes constitutes a limitation of the present review. Future research syntheses may benefit from specifically addressing these relational dimensions to offer a more comprehensive understanding of the multifaceted impact of infertility.

Another limitation is the small number of studies included in the meta‐analysis, which may have reduced the power of specific moderators as well as the overall statistical power of the meta‐analytic ES estimates. Moreover, the significant results of Egger's test and the asymmetric funnel plots suggest the presence of publication bias. The trim‐and‐fill method slightly increased the estimated ESs for both attachment anxiety and attachment avoidance; however, the significance, direction, and magnitude of the associations remained unchanged for both attachment dimensions. Therefore, when interpreting the results of this study, it is important to consider the small number of studies included in the meta‐analysis. Additionally, given the significance of the *Q* statistic, it is possible that other moderating variables not considered in this study could play a role.

Finally, it is important to highlight the absence of longitudinal studies that could track changes in psychological functioning over time, particularly considering the invasiveness of treatments and the uncertainty surrounding ART outcomes (Koert and Daniluk 2017). This lack of longitudinal data limits our ability to establish causal relationships between dimensions of attachment insecurity dimensions and aspects of distress or quality of life related to infertility. While theoretical models support the hypothesis that attachment represents a relatively stable trait dimension, it remains susceptible to modification in response to relational events (Mikulincer and Shaver 2016). In this context, longitudinal research could be especially valuable in clarifying whether and how infertility is perceived by some individuals as an unresolved trauma that impacts both their state of mind regarding attachment to their partner and, according to more recent models, their emotional regulation strategies (Schore and Schore 2008). Such studies would help deepen our understanding of the dynamic interplay between attachment and infertility‐related psychological distress. Further research could therefore be crucial for deepening our understanding of the role of attachment in relation to individual psychological correlates across the different stages of treatment.

### 4.2 Conclusions and Clinical Implications

The present systematic literature review and meta‐analysis examined the associations between romantic attachment within couple relationships and individual‐level psychological correlates related to infertility, underscoring the scientific relevance of this study domain. Both attachment anxiety and avoidance demonstrated significant relationships with infertility‐related distress and various dimensions of psychological well‐being. Infertility constitutes a substantial emotional burden for individuals and couples alike; for those high in attachment anxiety, this burden is compounded by persistent concerns about the stability of the relationship and the reliability of the partner (Mikulincer and Shaver 2016). Conversely, the emotional suppression characteristic of avoidant individuals may offer short‐term protection but tends to deteriorate under chronic stress, such as that experienced during infertility (Karveli et al. 2023).

Clinicians working with infertile couples have a unique opportunity to observe attachment dynamics in vivo. Even when therapy is conducted on an individual basis, the couple's relational dimension remains salient at a phantasmatic level. A nuanced understanding of these dynamics can help clarify each partner's subjective experience of infertility and their specific vulnerabilities. This highlights the clinical importance of recognizing the partner as an attachment figure (Shaver and Mikulincer 2009). Strengthening this bond can provide critical emotional support amid the challenges posed by infertility. Consequently, therapeutic interventions should remain adaptable, integrating individual and dyadic approaches as appropriate, while maintaining sensitivity to the attachment processes that influence coping and psychological adjustment. To inform effective interventions, further research is needed to clarify the roles of both attachment dimensions throughout the various phases of the infertility experience, including the coping mechanisms individuals employ when facing associated difficulties. Current evidence suggests that clinical work with infertile individuals should consider their partners' attachment styles, including their internal representations and interpersonal behaviors. Empirical findings reinforce the value of couple‐based interventions within infertility contexts, which have demonstrated beneficial effects on psychological well‐being (Stammer et al. 2002). Additionally, clinicians are encouraged to assess key areas highlighted in this review, such as perceived stress, depressive and anxious symptomatology, general well‐being, and body‐related concerns, to provide comprehensive care.

In conclusion, attachment theory offers a valuable framework for understanding the complex process of couple adaptation to infertility as a significant life stressor. Secure attachment, characterized by low levels of anxiety and avoidance, emerges as a protective factor against infertility‐related psychological difficulties (Iordăchescu et al. 2022; Mobeen and Dawood 2023).

## Supporting information

Appendix A. Search filter.

Appendix B_ID JMFT‐25‐0073 RV.

## Data Availability

The data that support the findings of this study are available on request from the corresponding author.
