# Supplementary material for: Attachment Dimensions and Infertility: Exploring Psychological Outcomes Through Systematic Review and Meta‐Analysis
Source: J Marital Fam Ther. 2025 Sep 8;51(4):e70073. doi: 10.1111/jmft.70073 (PMC12415507; doi:10.1111/jmft.70073)
Supplement: Supplementary file 1 — Appendix A. Search filter. [file JMFT-51-0-s002.docx]

**Appendix A**

*Search filter*

CONCEPT 1: ROMANTIC ATTACHMENT

“Attach*” OR “Experiences in close relationships” OR “Relationships style questionnaire”

CONCEPT 2: INFERTILITY

“Medically assisted procreation” OR “Fertilization” OR “Embryo transfer*” OR “Gamete intrafallopian transfer*” OR “Gamete intrafallopian tube transfer” OR “Intracytoplasmic sperm injection” OR “Infertil*” OR “Subfertility” OR “Subfecundity” OR “Steril*” OR “Infecundity” OR “Fertil*” OR “Insemination” OR “Childlessness” OR “Assisted reprod*” OR “Ovar* stimulation”
