# Supplementary material for: Attachment Dimensions and Infertility: Exploring Psychological Outcomes Through Systematic Review and Meta‐Analysis
Source: J Marital Fam Ther. 2025 Sep 8;51(4):e70073. doi: 10.1111/jmft.70073 (PMC12415507; doi:10.1111/jmft.70073)
Supplement: Supplementary file 2 — Appendix B_ID JMFT‐25‐0073 RV. [file JMFT-51-0-s001.docx]

**Appendix B**

*Studies investigating the association between romantic attachment and individual psychological outcomes in infertile patients***.**

**Table 1.** Studies investigating the relationship between attachment and infertility-related stress/quality of life.

|  |  |  | **Sample** | | | |  |  |  |  |  |
| --- | --- | --- | --- | --- | --- | --- | --- | --- | --- | --- | --- |
| **Authors (year)** | **Country** | **Study design** | **N°** | **Age (years)**  **M (SD)** | **Gender**  **(% males)** | **Sample status** | **Infertility assessment** | **Attachment instrument** | **Distress/QoL instruments** | **Other instruments** | **Main results** |
| * Donarelli et al. (2012) | Italy | Cross-sectional | 632 (316 couples) | Women 33.97 (4.84)  Men 37.01 (5.33) | 50% | Clinical | Primary infertility diagnosis and starting first IVF/ICSI treatment  (having had previous IVF or ICSI treatment was an exclusion criterion) | ECR (Experiences in Close Relationships Scale) | FPI (Fertility Problem Inventory) | STAI-S (State scale of State-Trait Anxiety Inventory) | *Significant positive associations between attachment insecurities (anxiety and avoidance) and infertility stress* *(overall score and all the FPI subscale scores, with the exception of rejection of childfree lifestyles) in both women and men.* |
| * Donarelli et al. (2016) | Italy | Cross-sectional | 770 (385 couples) | Women 34 (4.5)  Men 37 (5.1) | 50% | Clinical | Primary infertility diagnosis and starting initial ART treatment | ECR (Experiences in Close Relationships Scale) | FPI (Fertility Problem Inventory) | STAI-S (State scale of State-Trait Anxiety Inventory) | *Correlational analyses showed significant positive associations between both attachment anxiety and attachment avoidance and both women’s and men’s infertility stress; however, probability values were not reported because wives' and husbands' data were nested within the couple. Results from the Actor-Partner Interdependence Model (APIM) revealed, as the only actor effect, that wives' distress (infertility stress and state anxiety) was positively predicted by their own attachment avoidance.* |
| * Theodoridou et al. (2016) | Greece | Cross-sectional | 82 | 36.7 (4.3) | 0% | Clinical | Undergoing in vitro fertilization | ECR-R (Experiences in Close  Relationships-Revised) | FPI (Fertility Problem Inventory)  FertiQoL (Fertility Quality of Life Questionnaire) | - | *Significant positive associations between anxious attachment and infertility total stress and in the social, sexual and relationship concern subscales. Significant positive associations between avoidant attachment and the infertility-related relationship concern and negative association with the rejection of childfree lifestyle subscale.*  *Significant negative correlation between attachment anxiety and quality of life in the emotional, relational and social domains and in global infertility-related quality of life. Negative significant correlation between attachment avoidance and only the relationship quality subscale of FertiQoL.* |
| * Moura-Ramos et al. (2017) | Portugal | Cross-sectional | 90 (45 couples) | Women 32.04 (3.72)  Men 34.77 (5.22) | 50% | Clinical | Attending a  fertility center prior to ART treatments (IVF or ICSI) | AAS-R (Adult Attachment Scale—Revised) | FPI (Fertility Problem Inventory) Need For Parenthood subscale | WHOQoL-bref (World Health Organization Quality of Life abbreviated instrument) | *Significant positive association only between attachment avoidance and need for parenthood in both women and men.* |
| * Kalci et al. (2020) | Turkey | Cross-sectional | 105 | 32 (5.97) | 0% | Clinical | Attending an ART center and being eligible for  IVF | ECR (Experiences in Close Relationships Scale) | FPI (Fertility Problem  Inventory) | BSI (Brief Symptom Inventory) depression, anxiety and  somatization subscales; RIM (Resilience in Midlife Scale) | *Significant positive correlation between both attachment anxiety and attachment avoidance and infertility stress total score.* |
| * Molgora et al. (2020) | Italy | Cross-sectional | 480 (240 couples) | Women 36 (3.8)  Men 38.5 (5.5) | 50% | Clinical | Entering an ART program | ECR (Experiences in Close Relationships Scale) | ScreenIVF  helplessness, lack of acceptance and lack of perceived social support subscales | DAS (Dyadic Adjustment Scale) | *Attachment anxiety is positively associated with helplessness, lack of acceptance* *and lack of perceived social support in both women and men.*  *Attachment avoidance is positively associated with lack of perceived social support only in the men sample.* |
| * Renzi et al. (2020) | Italy | Cross-sectional | 88 | 37.42 (4.79) | 0% | Clinical | Undergoing ART for a fertility problem | ECR-R. (Experiences in Close  Relationships-Revised) | FertyQoL (Fertility Quality of Life) | CRI (Couple Relationship Inventory) | *Only attachment anxiety negatively correlated with FertyQoL overall score and its subscales, except for FertiQoL Relational subscale.* |
| * Iordăchescu et al. (2022) | Romania | Cross-sectional | 240 | 32.71 (4.85) | 0% | Clinical | Medical diagnosis of infertility (recruitment via social media) | ECR-S (Experiences in Close Relationships Scale - Short Form) | Difficulties Experienced Scale | STAI-Form Y (State-Trait Anxiety Inventory) | *Higher levels of experienced difficulties in case of anxious and avoidant attachment patterns than in case of the secure one.* |
| * Calvo et al. (2023) | Italy | Cross-sectional | 113 | 36.7 (6.0) | 0% | Clinical | Experiencing a diagnosis of infertility (female-related, male-related, combined or unexplained; recruitment via social media) | ECR-R (Experiences in Close  Relationships-Revised) | FPI-SF (Fertility Problem Inventory-Short Form) | BAS-2 (Body Appreciation Scale-2) | *Significant positive associations between attachment anxiety and attachment avoidance and infertility stress. Infertility stress was positively predicted only by attachment avoidance.*  *Positive body image mediated the association between attachment anxiety and infertility stress (greater levels of attachment anxiety were connected to lower levels of positive body image; higher levels of positive body image were related to lower levels of infertility stress).* |
| * Péloquin et al. (2024) | Canada | Cross-sectional | 174 (87 couples) | Women 32.12 (4.87)  Men 33.15 (5.12) | 50% | Clinical | Attending a fertility clinic for medical infertility | ECR-S (Experiences in Close Relationships Scale - Short Form) | FertiQoL (Fertility Quality of Life Questionnaire) | CIQ (Coping with Infertility Questionnaire) | *In the men sample, significant negative associations between both attachment anxiety and avoidance and quality of life; in the women sample only with attachment avoidance.*  *Path analyses based on APIM revealed that attachment anxiety was directly connected to reduced quality of life in women. In both women and men, attachment anxiety was connected to lower quality of life through their own higher*  *use of non-adaptive emotion-focused coping. Attachment avoidance in men was directly linked to their lower quality of life, while in women attachment avoidance was connected to their reduced quality of life through women’s and men’s lower use of adaptive emotion-focused coping.* |
| * Qu et al. (2024) | China | Cross-sectional | 448 (224 couples) | Women 30.5 (4.3)  Men 31.5 (4.9) | 50% | Clinical | Having received artificial insemination with donor  sperm | ECR (Experiences in Close Relationships Scale) | FPI (Fertility Problem Inventory) | - | *Women attachment avoidance and attachment anxiety positively predicted their own fertility pressure, as well as men attachment avoidance and attachment anxiety positively predicted their own fertility pressure.* |

**Table 2.** Studies investigating the relationship between attachment and general well-being.

|  |  |  | **Sample** | | | |  |  |  |  |  |
| --- | --- | --- | --- | --- | --- | --- | --- | --- | --- | --- | --- |
| **Authors (year)** | **Country** | **Study design** | **N°** | **Age (years)**  **M (SD)** | **Gender**  **(% males)** | **Sample status** | **Infertility assessment** | **Attachment instrument** | **Well-being instruments** | **Other instruments** | **Main results** |
| * Cassidy & McLaughlin (2016) | Ireland | Cross-sectional | 363 | - | 0% | Clinical | Undergoing an IVF cycle | Single-item measure (Brennan & Shaver, 1995) | GHQ-12 (General Health Questionnaire) | PSS-Fr and PSS-Fa Scales (Perceived Social Support Scales); Self-compassion Scale; Brief COPE; PMS (Parenthood Motivation Scale) | *Significant positive associations between both anxious and avoidant attachment and negative mental health; significant negative associations between both anxious and avoidant attachment and positive mental health. Secure attachment was negatively associated with negative mental health and positively with positive mental health.* |
| * Moura-Ramos et al. (2017) | Portugal | Cross-sectional | 90 (45 couples) | Women 32.04 (3.72)  Men 34.77 (5.22) | 50% | Clinical | Attending a  fertility center prior to ART treatments (IVF or ICSI) | AAS-R (Adult Attachment Scale—Revised) | WHOQoL-bref (World Health Organization Quality of Life abbreviated instrument) | FPI (Fertility Problem Inventory) Need For Parenthood subscale | *Both attachment anxiety and avoidance negatively predicted psychosocial well-being in both women and men.* |
| * McLaughlin & Cassidy (2019) | Ireland | Cross-sectional and longitudinal | 363 T1  305 T2 | 35.25 (2.5) (363 women at T1) | 0% | Clinical | T1: currently undergoing an IVF cycle  T2: one year after the first contact | Single-item measure (Brennan & Shaver, 1995) | GHQ-12 (General Health Questionnaire) positive mental health | PSS-Fr and PSS-Fa Scales (Perceived Social Support Scales); Self-compassion Scale; Brief COPE; PMS (Parenthood Motivation Scale) | *Positive mental health at both T1 (β=.110) and T2 (β=.125) was positively predicted by secure attachment (measured at T1).* |
| * Shlomo et al. (2019) | Israel | Cross-sectional | 177 women | 33.27 (5.31) | 0% | Clinical | Initial consultation at a fertility unit | ECR-S (Experiences in Close Relationships Scale - Short Form) | PANAS (Positive and Negative Affect) Satisfaction with Life Scale | NRI Network of Relationships Inventory | *Both attachment anxiety and attachment avoidance positively predicted negative affect, while attachment orientations did not make any significant contribution to positive affect.*  *Only attachment anxiety negatively predicted satisfaction in life.* |
| * Skvirsky et al. (2019) | Israel | Cross-sectional | 180 | 32.69 (5.24) | 0% | Clinical | Initial visit for ART | ECR-S (Experiences in Close Relationships Scale - Short Form) | PSS (Perceived Stress Scale) | MLQ (Meaning in Life Questionnaire); SDI (Self Disclosure Index) | *Higher attachment anxiety predicted higher perceived stress, while no significant association between attachment avoidance and perceived stress was detected. Self-disclosure to the mother mediated the relationship between attachment avoidance and perceived stress (higher avoidance was associated with lower self-disclosure which in turn was associated with higher perceived stress).* |

**Table 3.** Studies investigating the relationship between attachment and psychopathological symptoms.

|  |  |  | **Sample** | | | |  |  |  |  |  |
| --- | --- | --- | --- | --- | --- | --- | --- | --- | --- | --- | --- |
| **Authors (year)** | **Country** | **Study design** | **N°** | **Age (years)**  **M (SD)** | **Gender**  **(% males)** | **Sample status** | **Infertility assessment** | **Attachment instrument** | **Psychopathological symptoms instruments** | **Other instruments** | **Main results** |
| * Donarelli et al. (2012) | Italy | Cross-sectional | 632 (316 couples) | Women 33.97 (4.84)  Men 37.01 (5.33) | 50% | Clinical | Primary infertility diagnosis and starting first IVF/ICSI treatment  (having had previous IVF or ICSI treatment was an exclusion criterion) | ECR (Experiences in Close Relationships Scale) | STAI-S (State scale of State-Trait Anxiety Inventory) | FPI (Fertility Problem Inventory) | *Significant positive associations between attachment insecurities (anxiety and avoidance) and state anxiety in both women and men.* |
| * Donarelli et al. (2016) | Italy | Cross-sectional | 770 (385 couples) | Women 34 (4.5)  Men 37 (5.1) | 50% | Clinical | Primary infertility diagnosis and starting initial ART treatment | ECR (Experiences in Close Relationships Scale) | STAI-S (State scale of State-Trait Anxiety Inventory) | FPI (Fertility Problem Inventory) | *Correlational analyses showed significant positive associations between both attachment anxiety and attachment avoidance and both women’s and men’s state anxiety; however, probability values were not reported because wives' and husbands' data were nested within the couple. Results from the APIM model revealed, as the only actor effect, that wives' distress (infertility stress and state anxiety) was positively predicted by their own attachment avoidance.* |
| * Kalci et al. (2020) | Turkey | Cross-sectional | 105 | 32 (5.97) | 0% | Clinical | Attending an ART center and being eligible for  IVF | ECR (Experiences in Close Relationships Scale) | BSI (Brief Symptom Inventory) depression, anxiety and  somatization subscales | FPI (Fertility Problem  Inventory); RIM (Resilience in Midlife Scale) | *Significant positive correlation between both attachment anxiety and attachment avoidance and depressive and anxious symptoms. Significant positive correlation only between attachment avoidance (but not attachment anxiety) and somatization symptoms.* |
| * Molgora et al. (2020) | Italy | Cross-sectional | 480 (240 couples) | Women 36 (3.8)  Men 38.5 (5.5) | 50% | Clinical | Entering an ART program | ECR (Experiences in Close Relationships Scale) | ScreenIVF  anxiety and depression subscales | DAS (Dyadic Adjustment Scale) | *Attachment anxiety is positively associated with anxiety and depression in both women and men. In multiple linear regression, attachment anxiety positively predicts anxious symptoms only in the men sample.*  *Attachment avoidance is positively associated with anxiety and depression only in the men sample.* |
| * Salcuni et al. (2021) | Italy | Cross-sectional | 236 (118 couples) | Women 34.92 (3.98)  Men 37.45 (5.26) | 50% | Clinical | Undergoing for the first time the first stage of  ART procedures | ECR-R (Experiences in Close  Relationships-Revised) | GSI SCL-90-R (global severity  Index Symptom check list-90-revised) | BIAQ (Body image avoidance questionnaire); TAS-20 (Toronto Alexithymia  Scale-20); DAS (Dyadic Adjustment Scale) | *Significant positive association between attachment anxiety and global psychopathological severity in both women and men. Significant positive association between attachment avoidance and global psychopathological severity only in the men sample.* |
| * Iordăchescu et al. (2022) | Romania | Cross-sectional | 240 | 32.71 (4.85) | 0% | Clinical | Medical diagnosis of infertility | ECR-S (Experiences in Close Relationships Scale - Short Form) | STAI-Form Y (State-Trait Anxiety Inventory) | Difficulties Experienced Scale | *Higher levels of state-anxiety in case of anxious and avoidant attachment patterns than in case of the secure one.* |
| * Mobeen & Dawood (2023) | Pakistan | Cross-sectional | 80 | 31.5 (4.36) | 0% | Clinical | Infertility (primary or secondary) diagnosis Undergoing ART treatment for at least more than 2 years. | ASQ (Attachment Style Questionnaire) | Depression scale SCL-90 (Symptom check list-90) | RBI (Relationship Belief Inventory) | *Secure attachment negatively correlated*  *with depression, while anxious attachment was positively associated and significantly predicted depression.* |

**Table 4.** Studies investigating the relationship between attachment and the relationship with one's body.

|  |  |  | **Sample** | | | |  |  |  |  |  |
| --- | --- | --- | --- | --- | --- | --- | --- | --- | --- | --- | --- |
| **Authors (year)** | **Country** | **Study design** | **N°** | **Age (years)**  **M (SD)** | **Gender**  **(% males)** | **Sample status** | **Infertility assessment** | **Attachment instrument** | **Distress instruments** | **Body relationship instruments** | **Main results** |
| * Salcuni et al. (2021) | Italy | Cross-sectional | 236 (118 couples) | Women 34.92 (3.98)  Men 37.45 (5.26) | 50% | Clinical | Undergoing for the first time the first stage of  ART procedures | ECR-R (Experiences in Close  Relationships-Revised) | BIAQ (Body image avoidance questionnaire) | GSI SCL-90-R (global severity  Index Symptom check list-90-revised); TAS-20 (Toronto Alexithymia  Scale-20); DAS (Dyadic Adjustment Scale) | *Significant positive association between attachment anxiety and body image avoidance only in the women sample and between attachment avoidance and body image avoidance only in the men sample.* |
| * Calvo et al. (2023) | Italy | Cross-sectional | 113 | 36.7 (6.0) | 0% | Clinical | Experiencing a diagnosis of infertility (female-related, male-related, combined or unexplained) | ECR-R (Experiences in Close  Relationships-Revised) | BAS-2 (Body Appreciation Scale-2) | FPI-SF (Fertility Problem Inventory-Short Form) | *Attachment anxiety and attachment avoidance*  *negatively correlated with participants’ positive body image. Participants’ positive body image was negatively predicted only by attachment anxiety. Infertility stress mediated the association between attachment avoidance and lower levels of positive body image.* |
